# The crystal structure of the FAM134B–GABARAP complex provides mechanistic insights into the selective binding of FAM134 to the GABARAP subfamily

**DOI:** 10.1002/2211-5463.13340

**Published:** 2021-12-09

**Authors:** Junfeng Zhao, Zhiwei Li, Jianchao Li

**Affiliations:** ^1^ Division of Cell, Developmental and Integrative Biology School of Medicine South China University of Technology Guangzhou China; ^2^ Department of Otorhinolaryngology Guangzhou First People's Hospital School of Medicine South China University of Technology Guangzhou China

**Keywords:** Atg8, autophagy, FAM134B, GABARAP, LC3‐interacting region/Atg8‐interacting motif

## Abstract

The mammalian Atg8 family (Atg8s proteins) consists of two subfamilies: GABARAP and LC3. All members can bind to the LC3‐interacting region (LIR) or Atg8‐interacting motif and participate in multiple steps of autophagy. The endoplasmic reticulum (ER) autophagy receptor FAM134B contains an LIR motif that can bind to Atg8s, but whether it can differentially bind to the two subfamilies and, if so, the structural basis for this preference remains unknown. Here, we found that FAM134B bound to the GABARAP subfamily more strongly than to the LC3 subfamily. We then solved the crystal structure of the FAM134B–GABARAP complex and demonstrated that FAM134B used both its LIR core and the C‐terminal helix to bind to GABARAP. We further showed that these properties might be conserved in FAM134A or FAM134C. The structure also allowed us to identify the structural determinants for the binding selectivity. Our work may be valuable for studying the differential functions of GABARAP and LC3 subfamilies in ER phagy in future.

## Graphical abstract

The endoplasmic reticulum autophagy receptor FAM134B uses its Φ‐X1‐X2‐Ψ motif and C‐terminal α‐helix, or the “LC3‐interacting region core + C‐helix”, to bind to the GABARAP subfamily proteins preferentially and strongly.

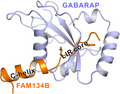

**Abbreviations**
ER: endoplasmic reticulum
GIM: GABARAP‐interacting motif
ITC: isothermal titration calorimetry
*K*_d_
: dissociation constant
LIR: LC3‐interacting region
PDB: protein data bank
SEC‐MALS: size‐exclusion chromatography coupled with multi‐angle light scattering

In eukaryotes, autophagy plays an important role in maintaining cellular homeostasis by degrading deleterious materials, such as protein aggregates, damaged organelles, and pathogens [1, 2, 3]. Autophagy is closely related to human diseases [4, 5]. Defects in autophagy may lead to neurodegenerative diseases, immune diseases, metabolic disorders, etc. [6, 7, 8, 9]. On the contrary, cancer cells can hijack autophagy pathways to provide sufficient nutrients and energy for their massive growth [10].

Autophagy is an evolutionarily conserved process involving multiple steps [2, 3, 11, 12, 13, 14]. It is controlled by a set of genes, including the Atg genes initially identified in yeast and EPG genes initially identified in *Caenorhabditis elegans* [15, 16]. Among them, Atg8 family proteins are engaged in nearly the entire process [17, 18, 19, 20]. For example, they can promote phagophore elongation and autophagosome formation [21, 22, 23]; they can help the fusion of autophagosome to lysosome [24, 25]; they can also recruit the autophagy receptors for selective target degradation [26, 27].

In mammals, Atg8 family contains two subfamilies, the GABARAP subfamily (including GABARAP, GABARAPL1, and GABARAPL2) and the LC3 subfamily (including LC3A, LC3B, and LC3C) [28]. They share high sequence similarity, and thus, they fold into similar three‐dimensional structures, that is, two N‐terminal α‐helix followed by a C‐terminal ubiquitin‐like domain [29, 30]. With these similar features, they can bind to proteins containing a motif called LC3‐interacting region (LIR) or Atg8‐interacting motif. This motif canonically contains a Φ‐X_1_‐X_2_‐Ψ motif, where Φ is an aromatic residue (W/Y/F), Ψ is an aliphatic residue (L/I/V), and X_1/2_ is any amino acid [31, 32]. These motifs normally bind to GABARAPs and LC3s with similar dissociation constants (*K*_d_) ranging from sub‐µm to a few µm. It was thought that these two subfamilies functioned redundantly in cells. However, more and more evidence shows that they play distinct roles in cells and are not interchangeable [23, 33, 34]. It is possible that some LIRs can differentiate these two subfamilies by binding to them with different affinities. Indeed, a study in 2017 showed that the Φ‐X_1_‐X_2_‐Ψ motif with a valine or isoleucine residue at the X_1_ position and a valine residue at the Ψ position ([W/F]‐[V/I]‐X_2_‐V, referred to as GABARAP‐interacting motif, GIM) preferably bound to GABARAPs 10‐ to 20‐fold stronger than to LC3s [35]. The discovery of LIR in giant ankyrin G (AnkG) revealed another possible mechanism [36, 37]. AnkG LIR uses an α‐helix (C‐helix) C‐terminal to the LIR core Φ‐X_1_‐X_2_‐Ψ motif to achieve extremely high affinity (a few nm) and high selectivity (it binds to GABARAPs 1000‐fold stronger than to LC3s). This “LIR core + C‐helix” mode has also been found in a series of LIR‐containing proteins including 440 kD ankyrin B, FYCO1, STX17, Ede1, Atg40, Sec62, RTN3, etc. [38, 39, 40, 41, 42]. Sequence‐based search has identified that FAM134B LIR might also fit this “LIR core + C‐helix” mode [37].

FAM134B, also called reticulophagy regulator 1, is a member of a family with sequence similarity 134. The family consists of three members, FAM134A/B/C, and studies on this family mainly focus on FAM134B. It can be anchored into the endoplasmic reticulum (ER) membrane via its transmembrane helices and bind to Atg8 family proteins via its LIR motif. Thus, it can serve as a selective autophagic receptor for ER autophagy (ER phagy) [43, 44, 45]. Dysfunction of FAM134B is associated with diseases such as inflammation, neuropathy, and cancer [46, 47, 48]. However, whether FAM134B LIR can preferentially bind to GABARAPs or LC3s and the structural basis for the preference remains elusive.

In this work, we studied the interactions between FAM134 family proteins and Atg8 family proteins in detail. We found that FAM134B preferred binding to the GABARAP subfamily, with about 10‐fold stronger affinity than to the LC3 subfamily. We then solved the crystal structure of FAM134B/GABARAP complex, revealing that FAM134B used the “LIR core + C‐helix” mode to bind to GABARAP and thus achieving the high affinity and high specificity. We also showed that the binding selectivity to GABARAP and the “LIR core + C‐helix” binding mode might be a general feature for the entire FAM134 family. Finally, our combined sequence and structural analyses provided insight into the structural determinants for the preferential binding. Our work might provide the biochemical foundation for further studying the differential functions of GABARAP and LC3 subfamilies in ER phagy.

## Results

### FAM134B_LIR interacts with GABARAP stronger than LC3A

**Fig. 1:**
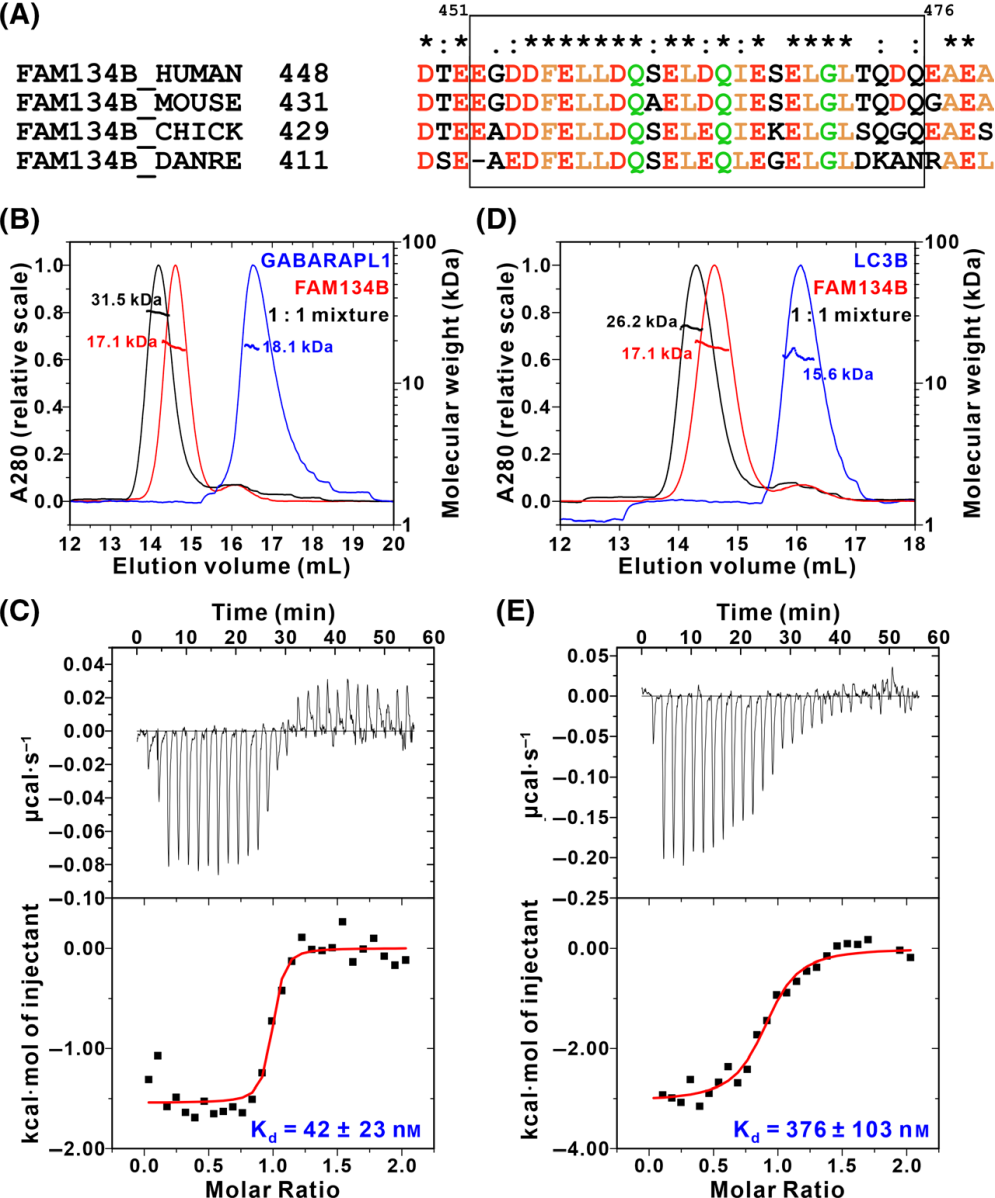
FAM134B LIR binds to GABARAP subfamily stronger than LC3 subfamily. (A) Sequence alignment showing that FAM134B LIR and its C‐terminal extension are highly conserved in vertebrates. The symbols above the sequences are defined as follows: an asterisk (*) indicates positions with a fully conserved residue; a colon (:) indicates conservation between groups of strongly similar properties; a period (.) indicates conservation between groups of weakly similar properties. Identical and highly similar residues are colored (yellow for hydrophobic residues, red for negatively charged residues, and green for other polar residues). (B, D) SEC‐MALS results (*n* = 3) showing that FAM134B strongly binds to GABARAPL1 (B) but binds to LC3B with lower affinity (D). (C, E) ITC‐based quantitative *K*_d_ measurements (*n* = 2) showing that the binding affinity between FAM134B and GABARAP (C) is about 10‐fold stronger than that between FAM134B and LC3A (E).

Sequence analysis reveals that FAM134B LIR (aa 451–476) is conserved among species in vertebrates (Fig. 1A). A previous study showed that deleting its C‐terminal reduced the binding to GABARAP dramatically, indicating that it also conformed to the “LIR core + C‐helix” mode [37]. We first used size‐exclusion chromatography coupled with multi‐angle light scattering (SEC‐MALS) to characterize its interaction with GABARAPL1 (a close paralog of GABARAP with > 80% sequence identity and it behaved much better in the SEC column). When mixing the two recombinant proteins at a 1 : 1 molar ratio, they were eluted with smaller elution volume and the measured molecular weight of 31.5 kDa was close to its theoretical molecular weight of 33.3 kDa, indicating that they formed a tight complex (Fig. 1B). Consistently, the measured *K*_d_ between FAM134B and GABARAP by isothermal titration calorimetry (ITC) was 42 nm (Fig. 1C), which was comparable to the previously reported 25 nm [37].

We next wanted to know whether FAM134B LIR binds to LC3s stronger or weaker than GABARAPs. Surprisingly, SEC‐MALS results showed that although FAM134B LIR can still form a complex with LC3B, the measured molecular weight (26.2 kDa) of the complex deviated from the theoretical one (33.8 kDa), suggesting that the binding affinity might be lower than that between FAM134B LIR and GABARAPL1 (Fig. 1D). In line with this, ITC showed that FAM134B LIR bound to LC3A with *K*_d_ around 376 nm (Fig. 1E), about 10‐fold weaker than that between FAM134B LIR and GABARAP.

### Overall structure of FAM134B_LIR/GABARAP complex

**Fig. 2:**
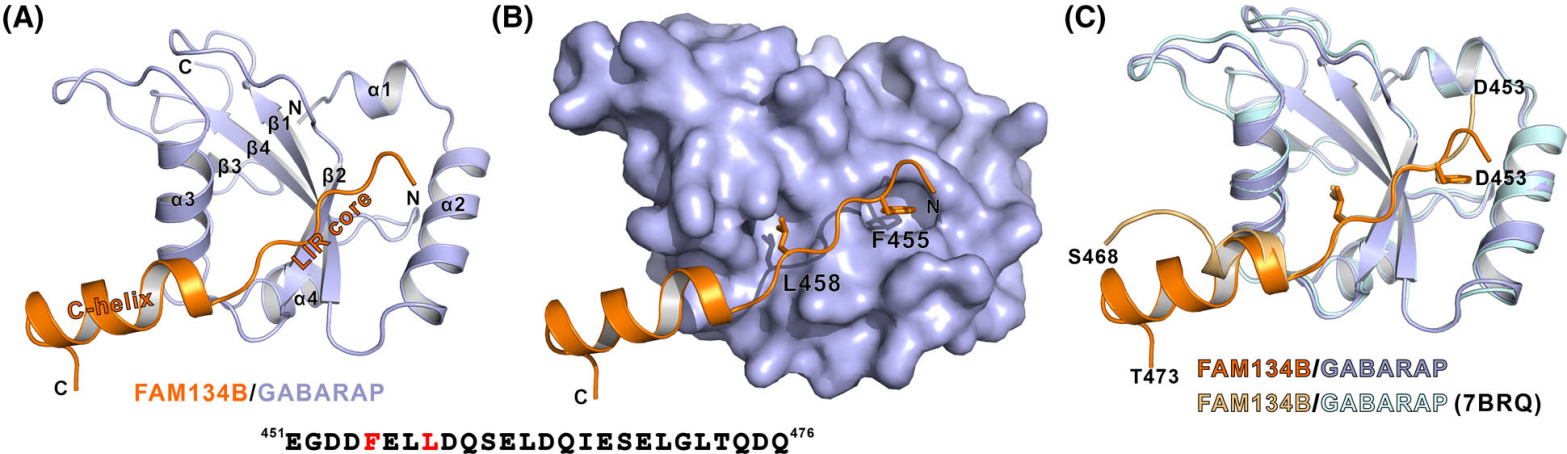
Overall structure of FAM134B/GABARAP complex. (A) Ribbon representations of the FAM134B/GABARAP complex crystal structure. The FAM134B is colored orange, and GABARAP is colored light blue. This coloring scheme is used throughout the whole paper except as otherwise indicated. (B) A combined surface (GABARAP) and ribbon/stick (FAM134B) representation showing the two residues at the Φ site and the Ψ site insert into the hydrophobic pockets of GABARAP. (C) Superposition of our solved structure and the recently reported FAM134B/GABARAP (FAM134B in gold and GABARAP in pale cyan) structure using fusion strategy (PDB: 7BRQ).

To understand the molecular basis of how FAM134B LIR interacts with GABARAP and the structural determinants of the differential binding, we tried to determine the high‐resolution structure of the complex. Using the chemically synthesized FAM134B LIR peptide and the recombinant GABARAP protein for crystallization, rod‐like crystals were obtained, and they were diffracted up to 2.85 Å (Table 1). The structure was then solved by molecular replacement methods. Agreeing with our expectation, the aromatic and aliphatic residues within the LIR core of FAM134B inserts into the two canonical hydrophobic pockets of GABARAP and its C terminus forms an α‐helix to interact with α3 of GABARAP (Fig. 2A,B).

**Table 1:** Statistics of X‐ray Crystallographic Data Collection and Model refinement. Numbers in parentheses represent the value for the highest resolution shell.

Data collection	
Data sets	FAM134B/GABARAP
Space group	*P3_2_21*
Wavelength (Å)	0.97915
Unit cell parameters (Å)	*a* = *b* = 73.250, *c* = 80.852 α = β=90°, γ = 120°
Resolution range (Å)	50–2.85 (2.90–2.85)
No. of unique reflections	6205 (310)
Redundancy	15.6 (15.9)
*I*/σ	19.6 (2.0)
Completeness (%)	99.9 (99.7)
*R*_merge_ ^a^ (%)	7.2 (69.4)
CC_1/2_ (last resolution shell)^b^	0.970

Structure refinement	
Resolution (Å)	50–2.85 (2.94–2.85)
*R*_cryst_ ^c^/*R*_free_ ^d^ (%)	18.59/24.38 (36.09/39.94)
rmsd bonds (Å)/angles (°)	0.007/1.422
Average *B* factor (Å^2^)^e^	71.09
No. of atoms	
Protein atoms	1142
Water	0
Ligands	0
No. of reflections	
Working set	5818 (558)
Test set	365 (48)
Ramachandran plot regions^e^	
Favored (%)	96.27
Allowed (%)	3.73
Outliers (%)	0

^a^ *R*_merge_ = S |*I_i_* − <*I*>|/S*I_i_*, where *I_i_* is the intensity of measured reflection and <*I*> is the mean intensity of all symmetry‐related reflections.

^b^ CC_1/2_ were defined by Karplus and Diederichs [63].

^c^ *R*_cryst_ = Σ||*F*_calc_| − |*F*_obs_||/Σ*F*_obs_, where *F*_obs_ and *F*_calc_ are observed and calculated structure factors.

^d^ *R*_free_ = Σ*_T_* ||*F*_calc_| − |*F*_obs_||/Σ*F*_obs_, where *T* is a test data set of about 5% of the total unique reflections randomly chosen and set aside prior to refinement.

^e^ *B* factors and Ramachandran plot statistics are calculated using MOLPROBITY [62].

During the period of our study, we noticed that a structure of FAM134B/GABARAP complex has been reported [41]. Comparing our structure to theirs, we found a few differences. First of all, the crystallization strategies were different. The crystallization for our structure determination was facilitated by mixing FAM134B peptide and GABARAP protein, whereas their structure was obtained by fusing FAM134B to the N terminus of GABARAP for successful crystallization. Moreover, the boundary of FAM134B we used for our structural study was aa 451–476 whereas they used aa 450–468. We believed that the longer C terminus and the direct mixing instead of fusion of the two proteins might better reflect the real state of how FAM134B C‐helix interact GABARAP, even though the resolution of our structure was lower. Indeed, by superimposing the two structures together, we can see that FAM134B C‐helix in our structure is a three‐turn helix and it turns inward and downward compared with their structure (Fig. 2C). The different conformations exhibited by the two structures might be due to the longer C‐helix and no steric hindrance induced by fusion of another GABARAP molecule to its C terminus.

### Detailed interactions between FAM134B_LIR and GABARAP

**Fig. 3:**
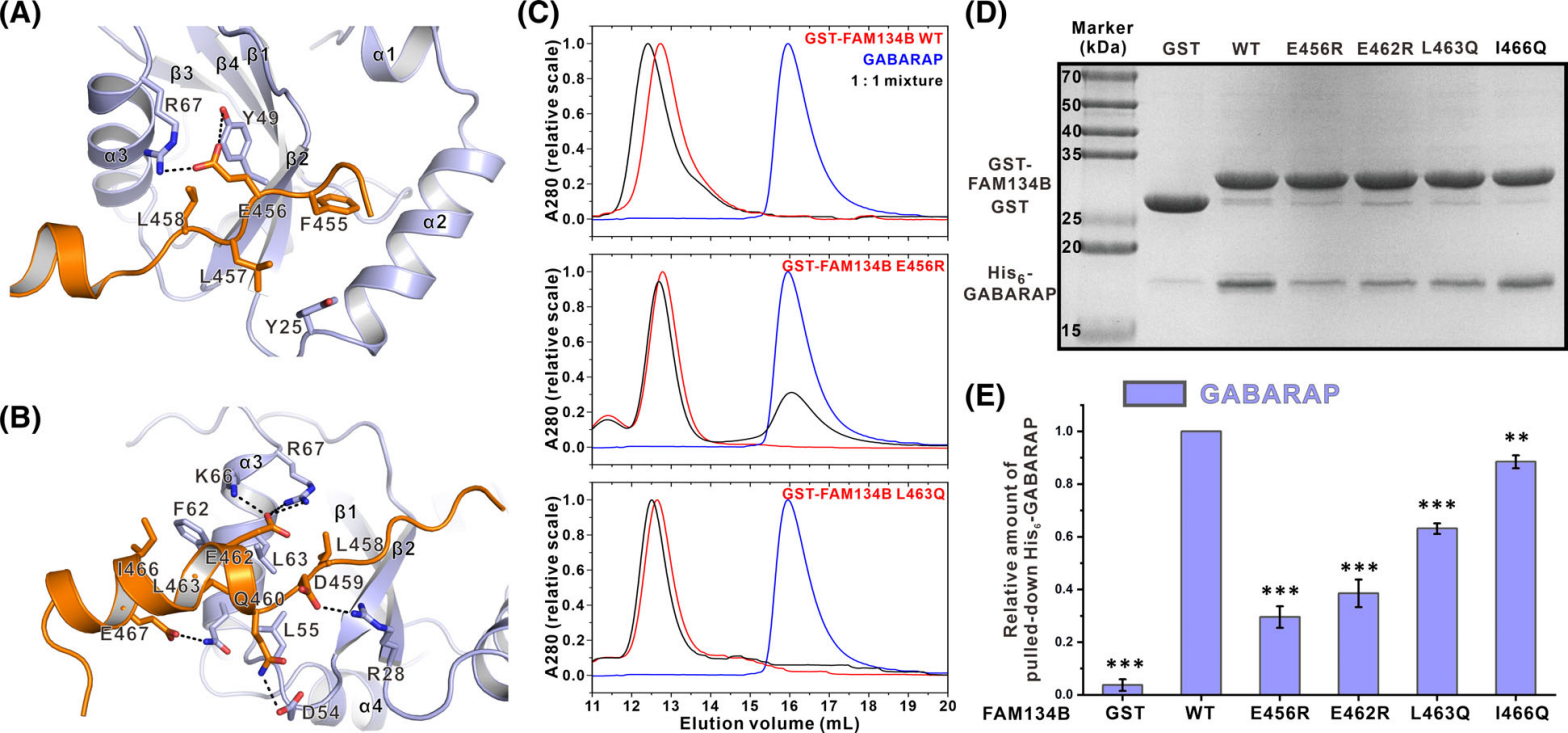
Detailed interactions between FAM134B and GABARAP. (A) E456 and L457 at the X_1_ and X_2_ sites are critical for FAM134B to bind to GABARAP as shown in the combined ribbon and stick representations. (B) The C‐helix of FAM134B is extensively involved in the interactions between FAM134B and GABARAP. (C) SEC results (*n* = 3) showing that the E456R mutation severely impaired the binding and L463Q mutation weakened the binding. (D) Representative pull‐down experiments (*n* = 4) showing that mutating critical residues in FAM134B weakened its interaction with GABARAP. (E) Quantification of the relative amount of GABARAP pulled down in the assays shown in panel *D*. The data were derived from four different batches of experiments, and the error bars were expressed as mean ± SEM and were analyzed with graphpad prism 9 using one‐way ANOVA followed by Tukey's multiple comparisons test; **P < 0.01; ***P < 0.001.

In addition to the insertion of F455 and L458 into the two hydrophobic pockets of GABARAP, the other residues in the LIR core of FAM134B also interact with GABARAP. Although FAM134B shows specificity to GABARAPs, it does not fit the pattern of the previously reported GIM [35]. The residue following the “Φ” site is not a Val or Ile but a Glu. This E456 has charge–charge interaction with R67 of GABARAP (Fig. 3A). Mutating E456 to a positively charged Arg severely impacted the binding, as shown by our SEC‐MALS and GST pull‐down experiments using purified proteins (Fig. 3C–E). L457, another residue flanked by the “Φ” site and “Ψ” site, also makes hydrophobic contact with Y25 of GABARAP (Fig. 3A).

The C‐helix of FAM134B is also extensively involved in the binding (Fig. 3B). D459 and Q460 proceeding the C‐helix form salt bridge and hydrogen bond with R28 and D54, respectively. E462 at the beginning of the C‐helix forms a salt bridge with R67 of GABARAP. Notably, this salt bridge has been observed at several other C‐helix containing LIR/Atg8s complexes, for example, AnkG/B and FYCO1 [37, 39]. Our structural observation was consistent with the previous mutagenesis result and our current GST pull‐down assay (Fig. 3D,E) that E462R mutation dramatically impaired FAM134B's binding to GABARAP. This salt bridge might be important for anchoring the C‐helix at the right position. FAM134B C‐helix is an amphiphilic helix, like the C‐helix of AnkG/B LIR. The hydrophobic side consisting of L463 and I466 forms hydrophobic interaction with F62 and L63, while the hydrophilic residue E467 forms a hydrogen bond with Q59. Again, the SEC‐MALS result showed that mutating L463 to a polar residue Gln mildly weakened the interaction (Fig. 3C). GST pull‐down assay also showed that slightly less amount of GABARAP was pulled down using GST‐FAM134B L463Q or I466Q mutant, compared with WT. In sum, our structure revealed how FAM134B used its LIR core and C‐helix to interact with GABARAP.

FAM134B belongs to the family with sequence similarity 134. The family contains another two members, FAM134A and FAM134C. Similarly, they can also use their LIRs to interact with both GABARAPs and LC3s [43]. Sequence alignment showed that both the LIR and the residues following in FAM134 family are highly conserved (Fig. S1A), suggesting that FAM134A and FAM134C might also conform to the “LIR core + C‐helix” mode. FAM134A and FAM134C likely exhibited similar binding selectivity to GABARAPs. To test our hypothesis, we first used SEC to qualitatively study the binding between FAM134A and Atg8s. When mixing FAM134A and GABARAPL1 at a 1 : 1 molar ratio, these two proteins were co‐eluted at a smaller elution volume, indicating that they interacted with each other. In contrast, when mixing at a 1 : 1 molar ratio, only a small portion of LC3B was co‐eluted with FAM134A and the majority of LC3B was eluted as a separated peak, suggesting that FAM134A bound to LC3B weakly (Fig. S1B,C). We failed to use ITC to quantitatively determine the *K*_d_ values for the bindings for direct comparison as the heat generation when titrating FAM134A to GABARAP/GABARAPL1 was too small to give reliable measurements (data not shown).

### Structural basis for the preferential binding between FAM134B and GABARAP

**Fig. 4:**
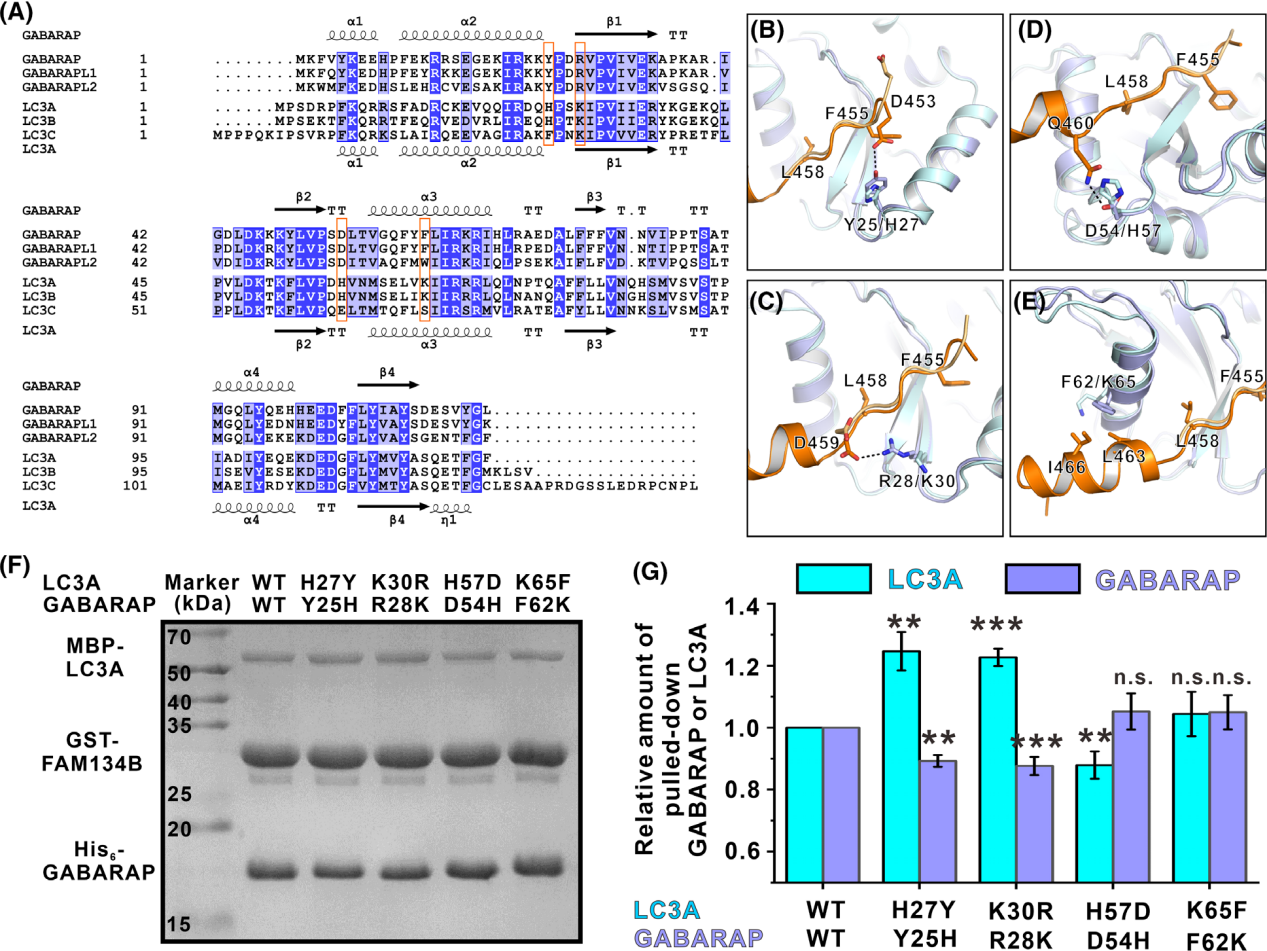
Combined sequence and structural analysis of the FAM134B/GABARAP binding selectivity. (A) Sequence alignment of human Atg8 family proteins. Residues that are identical and highly similar are shaded in blue and light blue, respectively. The secondary structure elements derived from the GABARAP structure are shown at the top and those from the LC3A structure are shown at the bottom of the alignment. Residues that might be critical for selectivity are highlighted in orange boxes. (B–E) Superposition of the FAM134B/GABARAP and the FAM134B/LC3B (light orange for FAM134B and pale cyan for LC3B) structure showing a few differences in the binding details. The F455 and L458 at the Φ site and the Ψ site are also shown to indicate the relative positions. (F) Representative competitive pull‐down experiments (*n* = 3) showing that the relative pulled‐down amount of GABARAP and LC3A were affected by exchange mutations of critical residues. (G) Quantification of the relative amount of GABARAP and LC3A pulled down in the assays shown in panel *F*. The data are derived from three different batches of experiments, and the error bars are expressed as mean ± SEM and were analyzed with graphpad prism 9 using one‐way ANOVA followed by Tukey's multiple comparisons test; ns: not significant, *P* > 0.05 **P < 0.01; ***P < 0.001.

We next wondered whether our structure can provide some implications of the reasons why FAM134B binds to GABARAPs stronger than LC3s. GABARAPs and LC3s originate from the same ancestor Atg8 and are structurally similar, but the two subfamilies evolved separately since the appearance of metazoan. As a result, the sequence identities among the same subfamily were over 80%, whereas the sequence identities between GABARAP/GABARAPL1 and LC3A/B were only 30–35%, as revealed by our analyses on human Atg8s sequences (Fig. 4A and Table S1). We anticipated that the structural determinants for the preferential binding to GABARAP might be the sites that were conserved in GABARAPs but not in LC3s.

We aligned the previously reported FAM134B/LC3A and our currently solved FAM134B/GABARAP structure together (Fig. 4B–E). By combining the sequence alignment of human Atg8 family proteins, we indeed found a few differences. First, Y25 of GABARAP forms a hydrogen bond with D453 of FAM134B. The corresponding residue in LC3A/B is a His and in LC3C is a Phe. In the FAM134B/LC3A structure, the H27 in LC3A does not interact with D453, as this residue points in the opposite direction (Fig. 4A,B). Moreover, D459, following the LIR core motif, forms a salt bridge with R28 in FAM134B/GABARAP structure. Although the corresponding residue of R28 in LC3s is also a positively charged residue Lys, it does not interact with D459 as shown in the FAM134B/LC3A structure (Fig. 4A,C). Furthermore, D54 of GABARAP forms a hydrogen bond with Q460. Q460 is not present in the FAM134B/LC3A structure, but it can be seen from the structure that H57, the counterpart of D54 in LC3A, is closer to the position of Q460. This might cause steric hindrance and weaken its binding to FAM134B (Fig. 4A,D). Finally, F62, located at GABARAP α3, makes hydrophobic contact with the amphiphilic C‐helix (Fig. 4A,E). It has already been shown that this Phe plays a critical role in dictating the binding selectivity between AnkG LIR and GABARAPs. It might also be important for FAM134B's preferential binding.

We tried to use GST pull‐down assay with purified recombinant proteins to verify the above observation. Agreeing with our analysis, mutating the four sites in LC3A to the corresponding residues of GABARAP (i.e., H27Y, K30R, H57D, and K65F) all enhanced the binding to GST‐FAM134B (Fig. S2). Then, we performed competition experiments to see whether the GABARAP/LC3A exchanged mutants can change the selectivity. For the WT group, the GST‐FAM134B could pull down more GABARAP and less LC3A (Fig. 4F), which is consistent with other biochemical results that FAM134B bound to GABARAP stronger than LC3A (Fig. 1). In two of the exchange mutations (H27Y/Y25H and K30R/R28K) groups, we could see more LC3A mutant and less GABARAP mutant being pulled down, compared with the WT group (Fig. 4F,G). For the H57D/D54H and K65F/F62K groups, we could not see more LC3A being pulled down in the presence of the GABARAP mutant (Fig. 4F,G). The reason might be that GABARAP carrying these two mutations also increased the binding to FAM134B. Nevertheless, the above pull‐down experiments verified that the four sites we identified prevent LC3A from strongly binding to FAM134B and thus contribute to the binding selectivity.

### General structural features of the “LIR core + C‐helix” binding mode

**Fig. 5:**
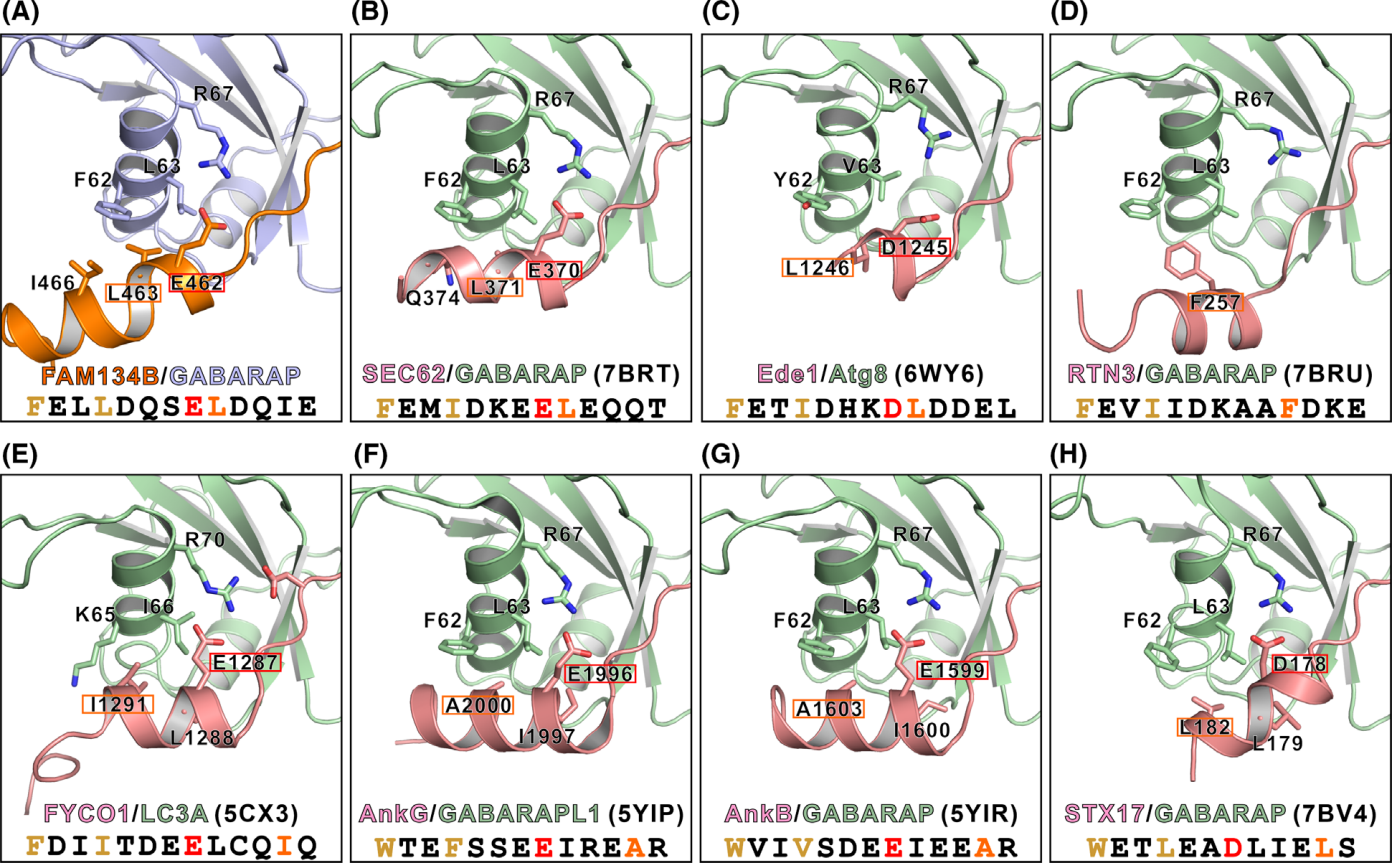
Summary of LIRs/Atg8s structures conforming to the “LIR core + C‐helix” binding mode. (A–H) Eight LIRs/Atg8s complex structures are selected and aligned together: our FAM134B/GABARAP (panel A), the previously reported SEC62/GABARAP (panel B), Ede1/Atg8 (panel C), RTN3/GABARAP (panel D), FYCO1/LC3A (panel E), AnkG/GABARAPL1 (panel F), AnkB/GABARAP (panel G), and STX17/GABARAP (panel H) structures. The sequences of these LIRs are also shown to indicate the relative positions of the Φ site and the Ψ site (highlighted in brown) in the LIR cores and the acid residue (highlighted in red) and hydrophobic residue (highlighted in orange) in the C‐helices.

More and more high‐resolution structures of the “LIR core + C‐helix” mode have now become available. Here, we try to summarize some general features revealed by these structures. We chose our currently solved FAM134B/GABARAP, and the previously reported AnkG/GABARAPL1, AnkB/GABARAP, STX17/GABARAP, RTN3/GABARAP, SEC62/GABARAP, FYCO1/LC3A, Ede1/Atg8 structures for detailed analysis [37, 39, 40, 41, 42]. By aligning all these structures together, we can see that these C‐helices vary in numbers of turns and orientations (Fig. 5). Despite these differences, there are a few common features. Firstly, except for RTN3, all these helices anchor to α3 of Atg8s via a salt bridge formed by an Arg (R67 in GABARAP/GABARAPL1, R70 in LC3A, and R70 in yeast Atg8) from Atg8s and a Glu or Asp (E462 in FAM134B, E1996 in AnkG, E1599 in AnkB, D178 in STX17, E370 in SEC62, E1287 in FYCO1, and D1245 in Ede1) from LIR‐containing proteins. In addition to the salt bridge, all C‐helices use a hydrophobic residue at essentially the same spatial position (L463 in FAM134B, A2000 in AnkG, A1603 in AnkB, L182 in STX17, F257 in RTN3, L371 in Sec62, I1291 in FYCO1, and L1246 in Ede1) to form hydrophobic interaction with α3 of Atg8s. The positions of the Glu or Asp in the primary sequences are mainly 4 residues C‐terminal to the Ψ site, that is, the +7 position (Fig. S3). The exceptional case is STX17, the Asp in which is only three residues apart, that is, the +6 position (Fig. S3). The relative positions between the hydrophobic residues and the acidic residues in the primary sequence can be grouped into two categories. In one category (FAM134B, SEC62, and Ede1), the two residues are precisely next to each other (+7 & +8 positions, Fig. S3), while in the other category (AnkG, AnkB, FYCO1, and STX17; although the +8 positions are also hydrophobic residues, they do not interact with α3 of Atg8s, Fig. 5E–H), the two residues are 4 residues apart (+7 & +11 positions, two neighboring turns in the three‐dimensional structures, Fig. S3). This variation may be due to the different orientations of the C‐helices. This explains why the consensus sequence derived from AnkG/B and FYCO1 failed to predict the LIRs in the other category. Nevertheless, these two common interactions together with other specific interactions facilitate the C‐helices binding.

It is worth noting that either of these two interactions can be missing if there are some compensatory interactions. For example, the C‐helix of RTN3 does not form a salt bridge with R67 of GABARAP. Instead, the hydrophobic interaction made by the two bulky residues F257 and F261 facilitates the anchoring of the C‐helix. Meanwhile, some LIRs with C‐terminal extensions also contain one or both of these elements. In the Atg40/Atg8 complex structure, D247 of Atg40 forms a salt bridge with R67 of Atg8. In the GABA_A_ receptor γ2 subunit (γ2‐GABA_A_R)/GABARAPL1 complex structure, the C413 of γ2‐GABA_A_R occupies the same position of the hydrophobic residues of other C‐helix containing LIRs [49].

## Discussion

Atg8 family proteins are central players throughout the entire autophagic process. To fulfill the diverse functions, they are able to bind to various proteins with the Φ‐X_1_‐X_2_‐Ψ motif. Growing evidence has shown that the GABARAP subfamily and the LC3 subfamily, or even each individual member, function distinctly in some specific process. Consistently, some of the LIR‐containing proteins, including but not limited to AnkG, FYCO1, PLEKHM1, PCM1, ULK1, γ2‐GABA_A_R, and FAM134 family proteins in this study, can differentially bind to the two subfamilies [35, 37, 39, 49, 50]. These proteins can be vaguely classified into two subgroups: one conforms to the previously identified GIM or [W/F]‐[V/I]‐X_2_‐V motif [35], and the other contains C‐terminal extension following the LIR core motif. We demonstrated here that FAM134 family belongs to the second group and the C‐terminal extension forms an α‐helix.

The “LIR core + C‐helix” binding mode was originally recognized in FYCO1 and AnkG and later found in a lot more LIR‐containing proteins, such as 440 kD ankyrin B, STX17, Ede1, Atg40, SEC62, RTN3, etc. The C‐helix mainly functions in two aspects. Firstly, it can greatly enhance the binding between LIR‐containing proteins and Atg8 family proteins. Take FAM134B in this study as an example. The involvement of the C‐helix enhances the binding affinity by more than 10‐fold. A similar enhancement effect was also found in AnkG/B [37]. Secondly, the C‐helix may contribute to the selective binding to GABARAPs or LC3s. The differences in hydrophobicity of Atg8s α3 have been noticed in an earlier study by Wirth et al. [50]. It is shown in our FAM134B/GABARAP and the previously solved AnkG/Atg8s structures that the C‐helices are amphiphilic, with their hydrophobic sides facing the α3. This feature allows the selection of different C‐helix containing LIR for strong binding.

Our FAM134B/GABARAP structure, along with other C‐helix containing LIR structures, reveals some common features of the C‐helices binding, but at the same time also highlights the diversities of the C‐helices. This implies the target recognition mechanisms of Atg8s are far beyond the current knowledge on the Φ‐X_1_‐X_2_‐Ψ motif. It is highly possible that residues N‐terminal or C‐terminal to the core motif contribute to the Atg8s/targets recognition with high affinity and high specificity. This also brings out new challenges on how to predict such targets.

The structure of FAM134B/GABARAP also underlines the importance of R67 in GABARAP (or the corresponding R70 in LC3A, R67 in yeast Atg8). On the one hand, as discussed above, R67 is responsible for making a salt bridge with acidic residues in C‐helices for their anchoring. On the other hand, R67 can also form a salt bridge with the acidic residue (E456 in FAM134B) at the X_1_ position of the Φ‐X_1_‐X_2_‐Ψ motif. Indeed, sequence analysis showed that nearly half of the LIRs contain an acidic residue at the X_1_ position. By inspecting some of the reported structures (e.g., RTN3/GABARAP, SEC62/GABARAP, STX17/GABARAP, Calreticulin/GABARAP, ATG14/GABARAPL1, ATG4B/GABARAPL1, γ2‐GABA_A_R/GABARAPL1, FYCO1/LC3A, FUNDC1/LC3B, TECPR2/LC3B, NEDD4/LC3B, Atg40/Atg8, Ede1/Atg8), we find that all the acidic residues, no matter Glu or Asp, invariably interact with the Arg in Atg8s [40, 41, 42, 49, 51, 52, 53, 54, 55, 56].

Last but not least, in contrast to the earlier study, we showed using our biochemical approaches (combination of ITC, SEC‐MALS, and GST pull‐down assays) in this study that FAM134 family proteins bound to GABARAPs stronger than to LC3s. Similarly, another ER‐phagy receptor, ATL3, has been reported to specifically bind to GABARAPs but not LC3s recently [57]. The differential binding to GABARAPs or LC3s by ER‐phagy receptors implies that these two subfamilies may have their distinct and specific functions in ER phagy. Further follow‐up investigations will help to address this remaining question.

## Conclusions

In summary, our biochemical and structural analysis demonstrated that FAM134B used the Φ‐X_1_‐X_2_‐Ψ motif and C‐terminal α‐helix, that is, the LIR core + C‐helix mode, to bind to the GABARAP subfamily proteins preferentially and strongly.

## Materials and methods

### Constructs, protein expression, and purification

The coding sequence of the human FAM134A fragment (Uniprot: Q8NC44, residues 488–513) was PCR‐amplified from synthetic oligonucleotides. The coding sequences of FAM134B (Uniprot: Q9H6L5), GABARAP (UniProt: Q9DCD6), GABARAPL1 (UniProt: Q8R3R8), LC3A (UniProt: Q91VR7), LC3B (UniProt: Q9CQV6) were generous gifts from M. Zhang (The Hong Kong University of Science and Technology). FAM134B peptide (residues 451–476) was synthesized from Shenzhen Pepbiotic Co. Ltd, Shenzhen, China. All constructs used for protein expression were cloned into pGEX‐6P‐1 or home‐modified pET32a vector. All point mutations were created using the QuikChange site‐directed mutagenesis kit and confirmed by DNA sequencing. Recombinant proteins were expressed in BL21 (DE3) *Escherichia coli* cells with induction by 0.1 mm IPTG at 16 °C. The N‐terminal Trx‐His_6_‐tagged proteins were purified using a Ni^2+^‐NTA agarose affinity column and GST‐tagged proteins were purified using Glutathione Sepharose, followed by size‐exclusion chromatography (Superdex 75 or Superdex 200 column from Cytiva, Marlborough, MA, USA) in a final buffer containing 100 mm NaCl, 50 mm Tris/HCl (pH 7.5), 1 mm DTT, and 1 mm EDTA. The purities and molecular weights were verified by SDS/PAGE and SEC‐MALS.

### Crystallography

The crystals of FAM134B/GABARAP were grown in a solution containing 0.1 m bis‐tris propane pH 8.0, 2.2 m dl‐Malic acid by hanging drop vapor diffusion methods at 16 °C. Crystals were soaked in the mother liquor containing additional 20% glycerol for cryoprotection before diffraction experiments. The diffraction data were collected at the wavelength of 0.97915 Å and temperature of 100 K at the Shanghai Synchrotron Radiation Facility BL18U1 beamline. Data were further processed and scaled using hkl3000 software [58].

The structure was solved by phaser software [59] using molecular replacement method with the apo‐form structure of GABARAP [protein data bank (PDB): 5YIR] as the search model. The model of FAM134B peptide was manually built according to the difference electron‐density map in coot [60]. Further model modifications and refinements were repeated alternatively using coot software and REFMAC5 software [61]. The final model was validated using MolProbity [62], and the statistics are shown in Table 1. The structure figures were made using pymol software (https://pymol.org/2/).

### Size‐exclusion chromatography coupled with multi‐angle light scattering

SEC‐MALS assays were performed on an ÄKTA Pure system (Cytiva) coupled with a Superose™ 12 10/300GL column, a static light scattering detector (miniDawn; Wyatt Technology), and a differential refractive index detector (Optilab; Wyatt Technology, Santa Barbara, CA, USA) in a column buffer composed of 100 mm NaCl, 50 mm Tris/HCl (pH 7.5), 1 mm DTT and 1 mm EDTA. Data were analyzed using ASTRA 7.3.1 (Wyatt Technology).

### Isothermal titration calorimetry

ITC experiments were performed on a VP‐ITC Microcal calorimeter (Malvern) at 25 °C. Titration buffer contained 50 mm Tris/HCl (pH 7.5), 100 mm NaCl, 1 mm DTT, and 1 mm EDTA. For a typical experiment, each titration point was obtained by injecting a 10 μL aliquot of GABARAP or LC3A sample (350 μm) into the cell containing Trx‐tagged FAM134B (35 μm) at a time interval of 120 s to ensure that the titration peak returned to the baseline. The titration data were analyzed using the one‐site binding model by Origin7.0.

### GST pull‐down assays

For GST pull‐down assays of FAM134B mutants or LC3A mutants, 1 µm GST‐tagged FAM134B or equimolar GST‐tagged FAM134B mutants (or GST as the negative control) was first incubated with the purified proteins of His‐tagged GABARAP or MBP‐tagged LC3A (or its mutants) for 1 h at 4 °C. For the competition experiments, 4 µm GST‐tagged FAM134B was first incubated with the purified proteins of 4.8 µm His‐tagged GABARAP (or its mutants) and 4.8 µm MBP‐tagged LC3A (or its mutants) for 1 h at 4 °C. Then, the 30 µL GSH‐Sepharose 4B slurry beads in PBS buffer were then incubated with the mixture for 30 min at 4 °C. After three times washing, the captured proteins were eluted by boiling, resolved by 10% SDS/PAGE, and detected by Coomassie blue staining. The Coomassie Brilliant Blue‐stained gels were analyzed by densitometry to determine the amount of pulled‐down proteins. Each experiment was repeated three times and data were analyzed with graphpad prism 9 (Graphpad Software, Inc., La Jolla, CA, USA) using one‐way ANOVA followed by Tukey's multiple comparisons test.

## Conflict of interest

The authors declare no conflict of interest.

## Author contributions

JZ and ZL performed experiments; all authors analyzed data; JL wrote the paper with inputs from other authors; and JL coordinated the project.

## Supporting information


**Fig. S1**. FAM134A binds to GABARAP subfamily stronger than to LC3 subfamily.
**Fig. S2**. FAM134B binds to LC3A mutants stronger than its WT.
**Fig. S3**. Sequence alignment of C‐helix containing LIRs shown in Fig. 5.
**Table S1**. Sequence identities among human Atg8 family proteins

## Data Availability

The atomic coordinates and the structure factors of FAM134B/GABARAP complex structure are available from the PDB under the accession code 7FB5.

## References

[1] Dikic I , Elazar Z . Mechanism and medical implications of mammalian autophagy. Nat Rev Mol Cell Biol. 2018;19:349–64. 29618831 10.1038/s41580-018-0003-4

[2] Zhang H , Baehrecke EH . Eaten alive: novel insights into autophagy from multicellular model systems. Trends Cell Biol. 2015;25:376–87. 25862458 10.1016/j.tcb.2015.03.001 PMC4475674

[3] Klionsky DJ , Abdel‐Aziz AK , Abdelfatah S , Abdellatif M , Abdoli A , Abel S , et al. Guidelines for the use and interpretation of assays for monitoring autophagy (4th edition). Autophagy. 2021;17:1–382. 33634751 10.1080/15548627.2020.1797280 PMC7996087

[4] Jiang P , Mizushima N . Autophagy and human diseases. Cell Res. 2014;24:69–79. 24323045 10.1038/cr.2013.161 PMC3879707

[5] Choi AM , Ryter SW , Levine B . Autophagy in human health and disease. N Engl J Med. 2013;368:651–62. 23406030 10.1056/NEJMra1205406

[6] Hubner CA , Dikic I . ER‐phagy and human diseases. Cell Death Differ. 2020;27:833–42. 31659280 10.1038/s41418-019-0444-0 PMC7206075

[7] Deng Z , Purtell K , Lachance V , Wold MS , Chen S , Yue Z . Autophagy receptors and neurodegenerative diseases. Trends Cell Biol. 2017;27:491–504. 28169082 10.1016/j.tcb.2017.01.001

[8] Menzies FM , Fleming A , Rubinsztein DC . Compromised autophagy and neurodegenerative diseases. Nat Rev Neurosci. 2015;16:345–57. 25991442 10.1038/nrn3961

[9] Levine B , Mizushima N , Virgin HW . Autophagy in immunity and inflammation. Nature. 2011;469:323–35. 21248839 10.1038/nature09782 PMC3131688

[10] Levy JMM , Towers CG , Thorburn A . Targeting autophagy in cancer. Nat Rev Cancer. 2017;17:528–42. 28751651 10.1038/nrc.2017.53 PMC5975367

[11] Nakatogawa H . Mechanisms governing autophagosome biogenesis. Nat Rev Mol Cell Biol. 2020;21:439–58. 32372019 10.1038/s41580-020-0241-0

[12] Hurley JH , Young LN . Mechanisms of autophagy initiation. Annu Rev Biochem. 2017;86:225–44. 28301741 10.1146/annurev-biochem-061516-044820 PMC5604869

[13] Galluzzi L , Baehrecke EH , Ballabio A , Boya P , Bravo‐San Pedro JM , Cecconi F , et al. Molecular definitions of autophagy and related processes. EMBO J. 2017;36:1811–36. 28596378 10.15252/embj.201796697 PMC5494474

[14] Bento CF , Renna M , Ghislat G , Puri C , Ashkenazi A , Vicinanza M , et al. Mammalian autophagy: how does it work? Annu Rev Biochem. 2016;85:685–713. 26865532 10.1146/annurev-biochem-060815-014556

[15] Tian Y , Li Z , Hu W , Ren H , Tian E , Zhao Y , et al. *C. elegans* screen identifies autophagy genes specific to multicellular organisms. Cell. 2010;141:1042–55. 20550938 10.1016/j.cell.2010.04.034

[16] Tsukada M , Ohsumi Y . Isolation and characterization of autophagy‐defective mutants of *Saccharomyces cerevisiae* . FEBS Lett. 1993;333:169–74. 8224160 10.1016/0014-5793(93)80398-e

[17] Johansen T , Lamark T . Selective autophagy: ATG8 family proteins. LIR motifs and cargo receptors. J Mol Biol. 2020;432:80–103. 31310766 10.1016/j.jmb.2019.07.016

[18] Wild P , McEwan DG , Dikic I . The LC3 interactome at a glance. J Cell Sci. 2014;127:3–9. 24345374 10.1242/jcs.140426

[19] Nakatogawa H , Ichimura Y , Ohsumi Y . Atg8, a ubiquitin‐like protein required for autophagosome formation, mediates membrane tethering and hemifusion. Cell. 2007;130:165–78. 17632063 10.1016/j.cell.2007.05.021

[20] Schaaf MB , Keulers TG , Vooijs MA , Rouschop KM . LC3/GABARAP family proteins: autophagy‐(un)related functions. FASEB J. 2016;30:3961–78. 27601442 10.1096/fj.201600698R

[21] Wu F , Watanabe Y , Guo XY , Qi X , Wang P , Zhao HY , et al. Structural basis of the differential function of the two *C. elegans* Atg8 homologs, LGG‐1 and LGG‐2, in autophagy. Mol Cell. 2015;60:914–29. 26687600 10.1016/j.molcel.2015.11.019

[22] Kraft C , Kijanska M , Kalie E , Siergiejuk E , Lee SS , Semplicio G , et al. Binding of the Atg1/ULK1 kinase to the ubiquitin‐like protein Atg8 regulates autophagy. EMBO J. 2012;31:3691–703. 22885598 10.1038/emboj.2012.225 PMC3442273

[23] Weidberg H , Shvets E , Shpilka T , Shimron F , Shinder V , Elazar Z . LC3 and GATE‐16/GABARAP subfamilies are both essential yet act differently in autophagosome biogenesis. EMBO J. 2010;29:1792–802. 20418806 10.1038/emboj.2010.74 PMC2885923

[24] Wang Z , Miao G , Xue X , Guo X , Yuan C , Wang Z , et al. The vici syndrome protein EPG5 Is a Rab7 effector that determines the fusion specificity of autophagosomes with late endosomes/lysosomes. Mol Cell. 2016;63:781–95. 27588602 10.1016/j.molcel.2016.08.021

[25] McEwan DG , Popovic D , Gubas A , Terawaki S , Suzuki H , Stadel D , et al. PLEKHM1 regulates autophagosome‐lysosome fusion through HOPS complex and LC3/GABARAP proteins. Mol Cell. 2015;57:39–54. 25498145 10.1016/j.molcel.2014.11.006

[26] Kirkin V , Lamark T , Sou YS , Bjorkoy G , Nunn JL , Bruun JA , et al. A role for NBR1 in autophagosomal degradation of ubiquitinated substrates. Mol Cell. 2009;33:505–16. 19250911 10.1016/j.molcel.2009.01.020

[27] Bjorkoy G , Lamark T , Brech A , Outzen H , Perander M , Overvatn A , et al. p62/SQSTM1 forms protein aggregates degraded by autophagy and has a protective effect on huntingtin‐induced cell death. J Cell Biol. 2005;171:603–14. 16286508 10.1083/jcb.200507002 PMC2171557

[28] Shpilka T , Weidberg H , Pietrokovski S , Elazar Z . Atg8: an autophagy‐related ubiquitin‐like protein family. Genome Biol. 2011;12:226. 21867568 10.1186/gb-2011-12-7-226 PMC3218822

[29] Paz Y , Elazar Z , Fass D . Structure of GATE‐16, membrane transport modulator and mammalian ortholog of autophagocytosis factor Aut7p. J Biol Chem. 2000;275:25445–50. 10856287 10.1074/jbc.C000307200

[30] Coyle JE , Qamar S , Rajashankar KR , Nikolov DB . Structure of GABARAP in two conformations: implications for GABA(A) receptor localization and tubulin binding. Neuron. 2002;33:63–74. 11779480 10.1016/s0896-6273(01)00558-x

[31] Noda NN , Ohsumi Y , Inagaki F . Atg8‐family interacting motif crucial for selective autophagy. FEBS Lett. 2010;584:1379–85. 20083108 10.1016/j.febslet.2010.01.018

[32] Birgisdottir AB , Lamark T , Johansen T . The LIR motif ‐ crucial for selective autophagy. J Cell Sci. 2013;126:3237–47. 23908376 10.1242/jcs.126128

[33] Manil‐Segalen M , Lefebvre C , Jenzer C , Trichet M , Boulogne C , Satiat‐Jeunemaitre B , et al. The *C. elegans* LC3 acts downstream of GABARAP to degrade autophagosomes by interacting with the HOPS subunit VPS39. Dev Cell. 2014;28:43–55. 24374177 10.1016/j.devcel.2013.11.022

[34] Grunwald DS , Otto NM , Park JM , Song D , Kim DH . GABARAPs and LC3s have opposite roles in regulating ULK1 for autophagy induction. Autophagy. 2020;16:600–14. 31208283 10.1080/15548627.2019.1632620 PMC7138202

[35] Rogov VV , Stolz A , Ravichandran AC , Rios‐Szwed DO , Suzuki H , Kniss A , et al. Structural and functional analysis of the GABARAP interaction motif (GIM). EMBO Rep. 2017;18:1382–96. 28655748 10.15252/embr.201643587 PMC5538626

[36] Tseng WC , Jenkins PM , Tanaka M , Mooney R , Bennett V . Giant ankyrin‐G stabilizes somatodendritic GABAergic synapses through opposing endocytosis of GABAA receptors. Proc Natl Acad Sci USA. 2015;112:1214–9. 25552561 10.1073/pnas.1417989112 PMC4313813

[37] Li J , Zhu R , Chen K , Zheng H , Zhao H , Yuan C , et al. Potent and specific Atg8‐targeting autophagy inhibitory peptides from giant ankyrins. Nat Chem Biol. 2018;14:778–87. 29867141 10.1038/s41589-018-0082-8

[38] Olsvik HL , Lamark T , Takagi K , Larsen KB , Evjen G , Overvatn A , et al. FYCO1 contains a C‐terminally extended, LC3A/B‐preferring LC3‐interacting region (LIR) motif required for efficient maturation of autophagosomes during basal autophagy. J Biol Chem. 2015;290:29361–74. 26468287 10.1074/jbc.M115.686915 PMC4705940

[39] Cheng X , Wang Y , Gong Y , Li F , Guo Y , Hu S , et al. Structural basis of FYCO1 and MAP1LC3A interaction reveals a novel binding mode for Atg8‐family proteins. Autophagy. 2016;12:1330–9. 27246247 10.1080/15548627.2016.1185590 PMC4968224

[40] Li Y , Cheng X , Li M , Wang Y , Fu T , Zhou Z , et al. Decoding three distinct states of the Syntaxin17 SNARE motif in mediating autophagosome‐lysosome fusion. Proc Natl Acad Sci USA. 2020;117:21391–402. 32817423 10.1073/pnas.2006997117 PMC7474698

[41] Mochida K , Yamasaki A , Matoba K , Kirisako H , Noda NN , Nakatogawa H . Super‐assembly of ER‐phagy receptor Atg40 induces local ER remodeling at contacts with forming autophagosomal membranes. Nat Commun. 2020;11:3306. 32620754 10.1038/s41467-020-17163-y PMC7335187

[42] Wilfling F , Lee CW , Erdmann PS , Zheng Y , Sherpa D , Jentsch S , et al. A selective autophagy pathway for phase‐separated endocytic protein deposits. Mol Cell. 2020;80:764–78.e7. 33207182 10.1016/j.molcel.2020.10.030 PMC7721475

[43] Khaminets A , Heinrich T , Mari M , Grumati P , Huebner AK , Akutsu M , et al. Regulation of endoplasmic reticulum turnover by selective autophagy. Nature. 2015;522:354–8. 26040720 10.1038/nature14498

[44] Mochida K , Oikawa Y , Kimura Y , Kirisako H , Hirano H , Ohsumi Y , et al. Receptor‐mediated selective autophagy degrades the endoplasmic reticulum and the nucleus. Nature. 2015;522:359–62. 26040717 10.1038/nature14506

[45] Bhaskara RM , Grumati P , Garcia‐Pardo J , Kalayil S , Covarrubias‐Pinto A , Chen W , et al. Curvature induction and membrane remodeling by FAM134B reticulon homology domain assist selective ER‐phagy. Nat Commun. 2019;10:2370. 31147549 10.1038/s41467-019-10345-3 PMC6542808

[46] Islam F , Gopalan V , Lam AK . RETREG1 (FAM134B): a new player in human diseases: 15 years after the discovery in cancer. J Cell Physiol. 2018;233:4479–89. 29226326 10.1002/jcp.26384

[47] Mo J , Chen J , Zhang B . Critical roles of FAM134B in ER‐phagy and diseases. Cell Death Dis. 2020;11:983. 33199694 10.1038/s41419-020-03195-1 PMC7670425

[48] Zhu L , Wang X , Wang Y . Roles of FAM134B in diseases from the perspectives of organelle membrane morphogenesis and cellular homeostasis. J Cell Physiol. 2021;236:7242–55. 33843059 10.1002/jcp.30377

[49] Ye J , Zou G , Zhu R , Kong C , Miao C , Zhang M , et al. Structural basis of GABARAP‐mediated GABAA receptor trafficking and functions on GABAergic synaptic transmission. Nat Commun. 2021;12:297. 33436612 10.1038/s41467-020-20624-z PMC7803741

[50] Wirth M , Zhang W , Razi M , Nyoni L , Joshi D , O'Reilly N , et al. Molecular determinants regulating selective binding of autophagy adapters and receptors to ATG8 proteins. Nat Commun. 2019;10:2055. 31053714 10.1038/s41467-019-10059-6 PMC6499816

[51] Thielmann Y , Weiergraber OH , Mohrluder J , Willbold D . Structural framework of the GABARAP‐calreticulin interface–implications for substrate binding to endoplasmic reticulum chaperones. FEBS J. 2009;276:1140–52. 19154346 10.1111/j.1742-4658.2008.06857.x

[52] Stadel D , Millarte V , Tillmann KD , Huber J , Tamin‐Yecheskel BC , Akutsu M , et al. TECPR2 cooperates with LC3C to regulate COPII‐dependent ER export. Mol Cell. 2015;60:89–104. 26431026 10.1016/j.molcel.2015.09.010

[53] Lv M , Wang C , Li F , Peng J , Wen B , Gong Q , et al. Structural insights into the recognition of phosphorylated FUNDC1 by LC3B in mitophagy. Protein Cell. 2017;8:25–38. 27757847 10.1007/s13238-016-0328-8 PMC5233613

[54] Qiu Y , Zheng Y , Wu KP , Schulman BA . Insights into links between autophagy and the ubiquitin system from the structure of LC3B bound to the LIR motif from the E3 ligase NEDD4. Protein Sci. 2017;26:1674–80. 28470758 10.1002/pro.3186 PMC5521552

[55] Skytte Rasmussen M , Mouilleron S , Kumar Shrestha B , Wirth M , Lee R , Bowitz Larsen K , et al. ATG4B contains a C‐terminal LIR motif important for binding and efficient cleavage of mammalian orthologs of yeast Atg8. Autophagy. 2017;13:834–53. 28287329 10.1080/15548627.2017.1287651 PMC5446077

[56] Birgisdottir AB , Mouilleron S , Bhujabal Z , Wirth M , Sjottem E , Evjen G , et al. Members of the autophagy class III phosphatidylinositol 3‐kinase complex I interact with GABARAP and GABARAPL1 via LIR motifs. Autophagy. 2019;15:1333–55. 30767700 10.1080/15548627.2019.1581009 PMC6613885

[57] Chen Q , Xiao Y , Chai P , Zheng P , Teng J , Chen J . ATL3 Is a Tubular ER‐Phagy receptor for GABARAP‐mediated selective autophagy. Curr Biol. 2019;29:846–55.e6. 30773365 10.1016/j.cub.2019.01.041

[58] Otwinowski Z , Minor W . Processing of X‐ray diffraction data collected in oscillation mode. Methods Enzymol. 1997;276:307–26. 10.1016/S0076-6879(97)76066-X 27754618

[59] Mccoy AJ , Grosse‐Kunstleve RW , Adams PD , Winn MD , Storoni LC , Read RJ . Phaser crystallographic software. J Appl Crystallogr. 2007;40:658–74. 19461840 10.1107/S0021889807021206 PMC2483472

[60] Emsley P , Lohkamp B , Scott WG , Cowtan K . Features and development of Coot. Acta Crystallogr D Biol Crystallogr. 2010;66:486–501. 20383002 10.1107/S0907444910007493 PMC2852313

[61] Murshudov GN , Skubak P , Lebedev AA , Pannu NS , Steiner RA , Nicholls RA , et al. REFMAC5 for the refinement of macromolecular crystal structures. Acta Crystallogr D Biol Crystallogr. 2011;67:355–67. 21460454 10.1107/S0907444911001314 PMC3069751

[62] Chen VB , Arendall WB , Headd JJ , Keedy DA , Immormino RM , Kapral GJ , et al. MolProbity: all‐atom structure validation for macromolecular crystallography. Acta Crystallogr D Biol Crystallogr. 2010;66:12–21. 20057044 10.1107/S0907444909042073 PMC2803126

[63] Karplus PA , Diederichs K . Linking crystallographic model and data quality. Science. 2012;336:1030–3. 22628654 10.1126/science.1218231 PMC3457925

