# Supplementary material for: The crystal structure of the FAM134B–GABARAP complex provides mechanistic insights into the selective binding of FAM134 to the GABARAP subfamily
Source: FEBS Open Bio. 2021 Dec 9;12(1):320–31. doi: 10.1002/2211-5463.13340 (PMC8727931; doi:10.1002/2211-5463.13340)
Supplement: Supplementary file 1 — Fig. S1. FAM134A binds to GABARAP subfamily stronger than to LC3 subfamily. Fig. S2. FAM134B binds to LC3A mutants stronger than its WT. Fig. S3. Sequence alignment of C‐helix containing LIRs shown in Fig. 5. Table S1. Sequence identities among human Atg8 family proteins [file FEB4-12-320-s001.docx]

Table S1 Sequence identities among human Atg8 family proteins

| Sequence identity (%) | GABA-RAP | GABA-RAPL1 | GABA-RAPL2 | LC3A | LC3B | LC3C |
| --- | --- | --- | --- | --- | --- | --- |
| GABARAP | 100 |  |  |  |  |  |
| GABARAPL1 | 87.1 | 100 |  |  |  |  |
| GABARAPL2 | 57.8 | 61.2 | 100 |  |  |  |
| LC3A | 30.7 | 32.5 | 41.7 | 100 |  |  |
| LC3B | 31.3 | 35.2 | 37.5 | 82.5 | 100 |  |
| LC3C | 38.6 | 34.2 | 43 | 59.2 | 55 | 100 |

**Figure S1**


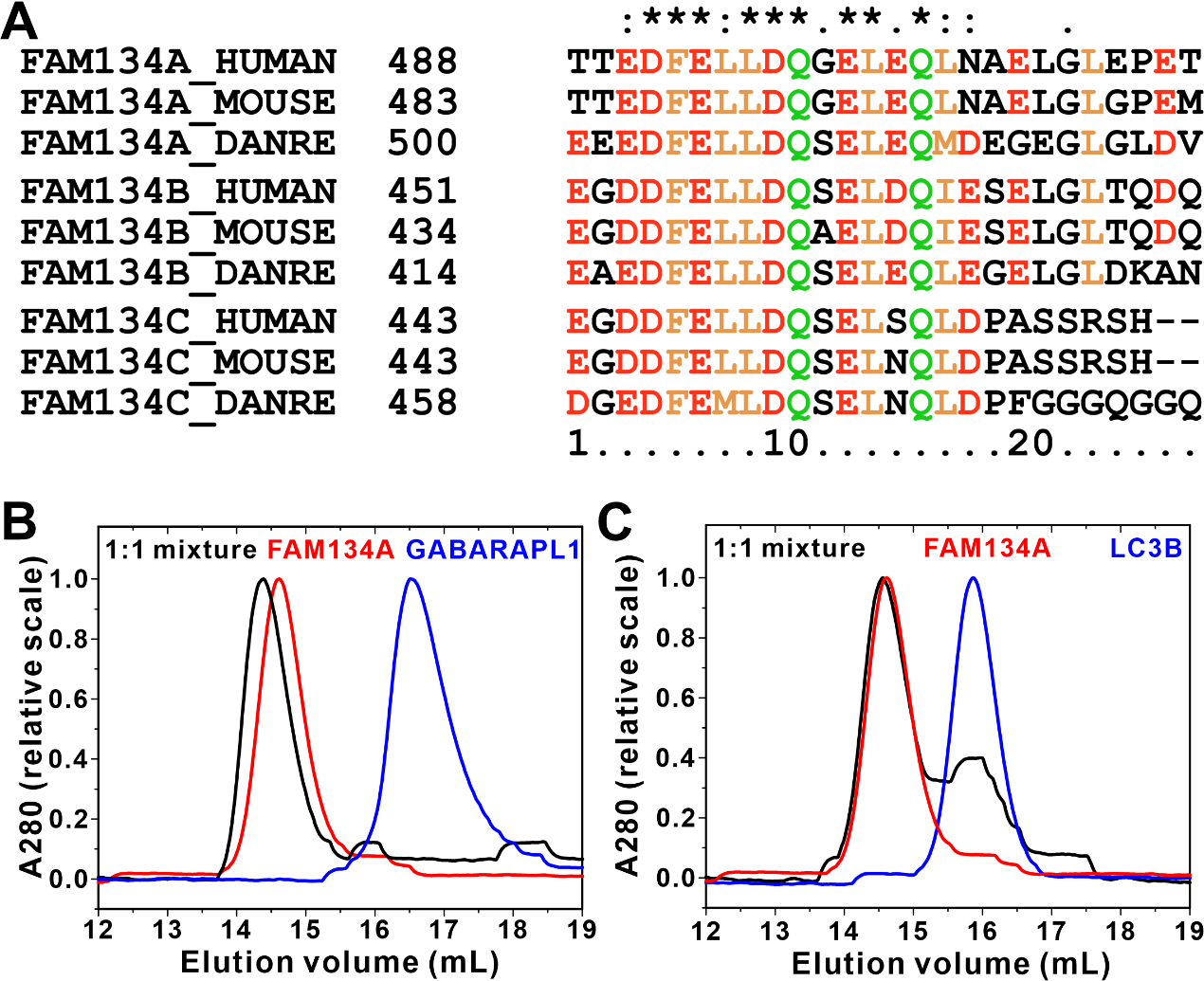


**Figure S1: FAM134A binds to GABARAP subfamily stronger than to LC3 subfamily.**

(A) Sequence alignment of the LIR of vertebrate FAM134 family proteins. (B&C) SEC results showing that FAM134A strongly binds to GABARAPL1 (B) but only weakly binds to LC3B (C).

**Figure S2**


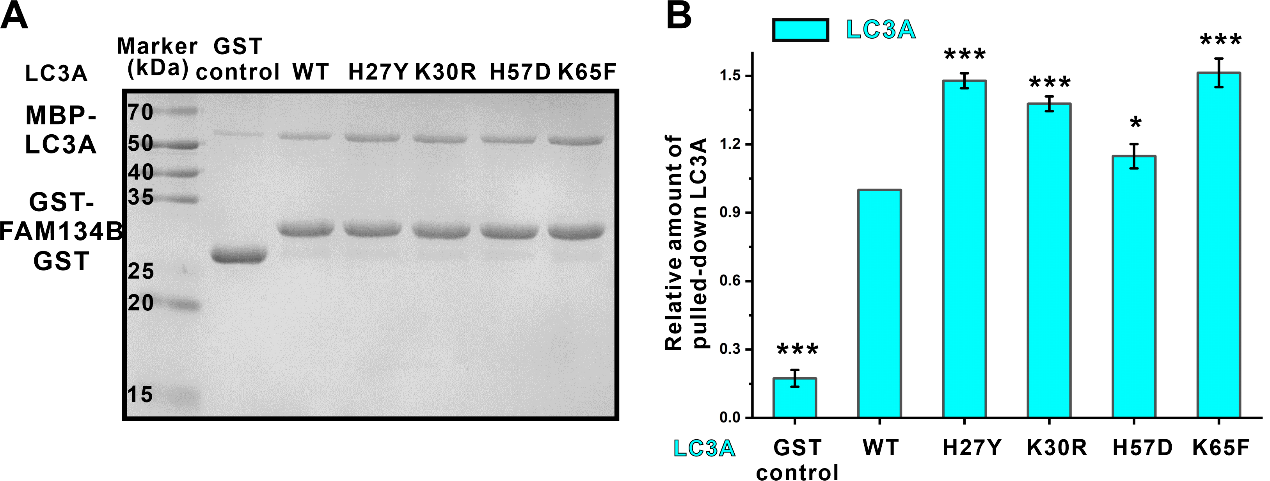


**Figure S2: FAM134B binds to LC3A mutants stronger than its WT.**

(A) Representative pull-down experiments (n=3) showing that mutating critical residues in LC3A enhanced its interaction with FAM134B. (B) Quantification of the amount of LC3A (or its mutants) pulled down in the assays shown in panel *A*. The data are derived from three different batches of experiments, and the error bars are expressed as mean ± SEM and were analyzed with GraphPad Prism 9 using one-way ANOVA followed by Tukey's multiple comparisons test. *: P < 0.05; ***: P < 0.001.

**Figure S3**


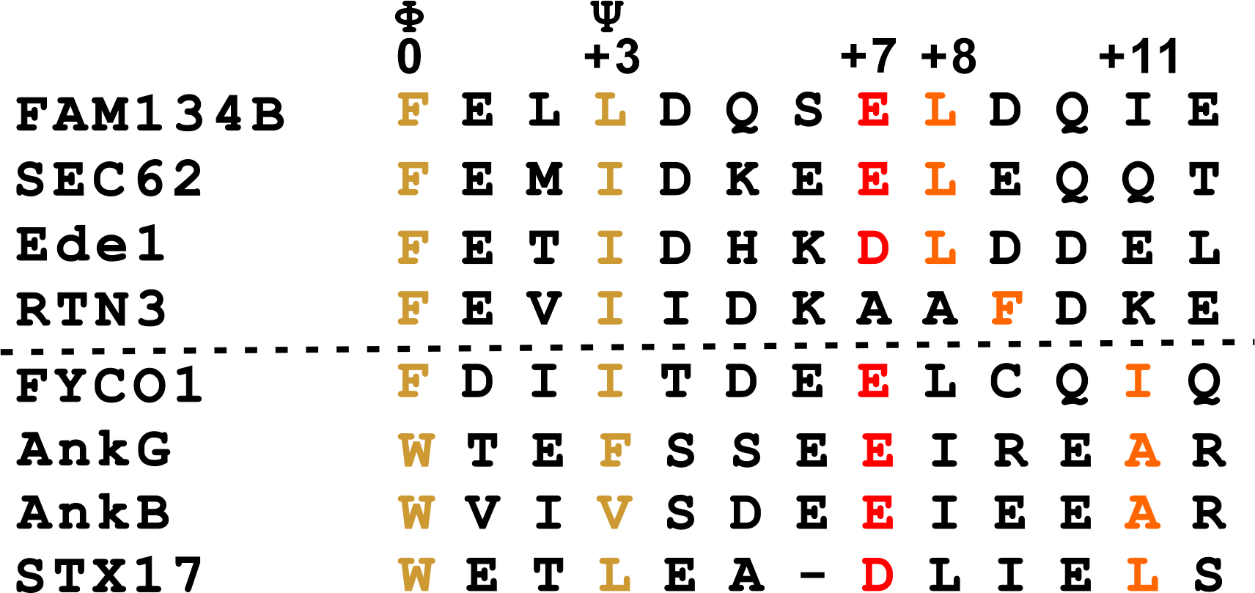


**Figure S3: Sequence alignment of C-helix containing LIRs shown in Figure 5.**

The Φ site and the Ψ site (highlighted in brown) in the LIR cores are denoted as the “0” and “+3” positions. The acid residues (highlighted in red) are located at the “+7” position (except for RTN3 and STX17). The hydrophobic residues (highlighted in orange) that interact with α3 of Atg8s are located at “+8” positions for category I LIRs (except for RTN3) and at “+11” positions for category II LIRs.
